# Smells Like Teen Spirit—A Model to Generate Laundry-Associated Malodour In Vitro

**DOI:** 10.3390/microorganisms9050974

**Published:** 2021-04-30

**Authors:** Marc-Kevin Zinn, Marco Singer, Dirk Bockmühl

**Affiliations:** 1 Faculty of Life Sciences, Rhine-Waal University of Applied Sciences, 47533 Kleve, Germany; marc-kevin.zinn@hochschule-rhein-waal.de; 2 Symrise AG, 37603 Holzminden, Germany; marco.singer@symrise.com

**Keywords:** malodour, GC, sensors, *Micrococcus luteus*, *Staphylococcus hominis*, *Corynebacterium jeikeium*

## Abstract

Although malodour formation on textiles and in washing machines has been reported to be a very relevant problem in domestic laundry, the processes leading to bad odours have not been studied intensively. In particular, the smell often described as “wet-and-dirty-dustcloth-like malodour” had not been reproduced previously. We developed a lab model based on a bacterial mixture of *Micrococcus luteus*, *Staphylococcus hominis*, and *Corynebacterium jeikeium*, which can produce this odour type and which might allow the detailed investigation of this problem and the development of counteractions. The model uses bacterial strains that have been isolated from malodourous textiles. We could also show that the three volatile compounds dimethyl disulfide, dimethyl trisulfide, and indole contribute considerably to the “wet-fabric-like” malodour. These substances were not only found to be formed in the malodour model but have already been identified in the literature as relevant malodourous substances.

## 1. Introduction

Malodour formation in domestic laundry has become a relevant problem for many consumers, especially since wash temperatures have steadily decreased [1,2,3,4]. Although there are several types of odours that can be associated with textiles [5], this study focuses on the “wet-and-dirty-dustcloth-like” or “wet fabric” malodour [6,7,8]. This problem may occur with laundry when it is hung to dry indoors or even with textiles that have already been dried and stored in damp conditions [3,6,8]. 

Multiple factors have been shown to impact the formation of malodour. Apart from human skin and clothing, the washing machine can be considered an important source of water-borne bacteria [5], whereas human skin serves as a reservoir for several members of the transient and resident skin microbiota. Some studies report microbial counts on textiles of up to 10^6^ cfu/cm^2^, mainly comprising the genera *Staphylococcus*, *Corynebacterium,* and the group Betaproteobacteria [9,10,11]. Thus, it must be assumed that the development of body odour is one of the factors influencing the formation of laundry associated malodour as well. Troccaz et al. showed that *Corynebacterium tuberculostearicum*, *Staphylococcus hominis,* and *Anaerococcus* spp. are main species that contribute to the formation of body odour [12]. For the typical volatile compounds related to sweaty odour, 3-methyl-2-hexenoic acid (3M2H), and 3-hydroxy-3-methylhexanoic acids, Corynebacteria have been shown to be a major source by releasing these substances in a reaction using the enzyme *N*-acylglutamine aminoacylase [13,14,15].

Although the formation of body odours occurs directly on the skin, textiles might play an important role in the formation and retention of the odourous substances. Several volatile compounds such as short-chain fatty acids and branched-chain fatty acids can be found on unwashed clothing in a textile-dependent distribution [16,17]. However, various odourous compounds can also be detected after washing. Besides fatty acids such as 3–methylbutanoic acid [3], steroids (e.g., 5–a–androst–2–en–17–one) [3], ketones (e.g., 1–octen–3–one) [3], esters (e.g., ethyl–2–methylpropanoate) [3], aldehydes (e.g., hexanal) [16], and alcohols (e.g., oct–1–en–3–ol) [18] were also found. In addition to these compounds, sulfuric compounds such as dimethyl disulfides (DMDS) and dimethyl trisulfides (DMTS) have shown to be present on odourous textiles as well [19,20] Some studies have also tried to analyse microorganisms present on malodourous textiles and have predominantly identified *Staphylococcus* sp. and *Micrococcus* sp., and in smaller numbers, *Bacillus* sp., *Enterobacteriaceae*, and *Acinetobacter* sp. [17,21,22].

In addition, the washing machine may be an underestimated factor that can influence the formation of textile malodour. Honisch et al. systematically analysed the influences of temperature, time, and detergent on the antimicrobial efficacy of laundering in household washing machines [23]. While it has been shown that temperatures of 50 °C and above result in sufficient bacterial reduction on contaminated textiles, these temperatures might not be used in domestic laundering on a regular basis [24,25], and more importantly may not be reached everywhere in the washing machine [2,26,27]. Moreover, the washing machine provides a good habitat for microbial growth. Biofilms in washing machines have been described in several studies and appear to consist mainly of the genera *Acinetobacter*, *Bacillus*, *Brevundimonas*, *Micrococcus*, *Staphylococcus,* and *Pseudomonas* [3,8,28]. On textiles left in the washing machine overnight, *Pseudomonas* spp. have been shown to be present [28].

Besides the growing knowledge about the factors responsible for laundry-associated malodour, the investigation of this problem remains difficult, since the formation of malodourous substances is obviously impacted by many parameters, often randomly leading to malodour. While some experimental setups for the investigation of sweaty odours exist [29,30,31], the “wet-and-dirty-dustcloth-like” odour has hardly been approached experimentally. To overcome this drawback, we developed a laboratory model for the examination of laundry-associated malodour of this type, which also allows for the investigation of possible counteractions. 

## 2. Materials and Methods

### 2.1. Preparation of the Swatches

First, 1 g of beef tallow (local supplier) and 1.5 g of TEGO Care PS (Evonik Industries AG, Essen, Germany) (emulsifier) were weighed and added to a 50 mL tube with 25 mL of 0.9% NaCl (AppliChem GmbH, Darmstadt, Germany). The components were then heated in a water bath at 80 to 90 °C for 30 min. This was followed by cooling to 40 °C in a shaking incubator (20 °C, 200 rpm) for 15 min. For further use, 10 mL of the beef tallow mixture was used for each microorganism.

Cultures grown on solid culture media were used to prepare the swatches. For this procedure, three fully grown agar plates were completely released from the microorganisms and placed in 30 mL tryptic soy broth (TSB) (Merck KGaA, Darmstadt, Germany), which was in a 50 mL Erlenmeyer flask. Incubation was performed in a shaker incubator at 200 rpm for 24 h at 37 °C. The overnight culture was transferred to a sterile 50 mL tube and then centrifuged at 4800 rpm for 10 min. The supernatant was decanted, and the pellet was resuspended in 10 mL of 0.9% NaCl. To wash the pellet, the resuspended solution was centrifuged at 4800 rpm for 10 min. The supernatant was decanted and the pellet was resuspended in 10 mL of the beef tallow mixture. Subsequently, 1 mL of each microorganism–bovine tallow solution was first pipetted onto a 2 × 2 cm textile (cotton) wfk10A, wfk-Testgewebe GmbH (Brüggen, Germany) located in a Petri dish. Furthermore, the respective microorganism combinations (Table 1 and Table 3) were prepared in 1.5 mL reaction tubes. For the combinations of the three bacteria, 333 μL of each microorganism–cattle tallow solution was pipetted into the reaction tube, so that a volume of 999 µL of the microorganism combinations could be pipetted onto the textiles. For incubation, the prepared samples were stored in a constant climate chamber (HPP110; Memmert GmbH and Co. KG, Schwabach, Germany) at 27 °C and 84% RH.

### 2.2. Gas Chromatography

**Figure 1:**
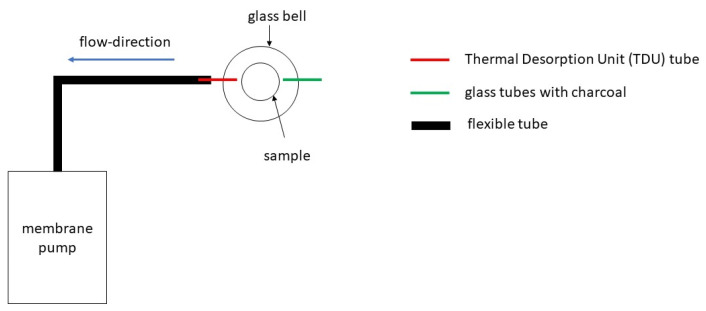
Setup for the sampling with assistance of the Scent Trap Kit.

The samples were taken using a Scent Trap Kit (Symrise AG, Holzminden, Germany). This kit takes the sample from the headspace of the sample to be analysed. The kit consists of a diaphragm pump, a tube, several thermal desorption unit (TDU) tubes filled with Tenax (Gerstel GmbH and Co. KG, Mülheim an der Ruhr, Germany), a glass bell jar, and a glass tube filled with activated carbon. The membrane pump draws gas from the sample headspace through the TDU tube. The glass tube with charcoal prevents outside odours entering the sample. The entire setup is shown in Figure 1.

The most important gas chromatographic parameters are listed in Table 2. A detailed summary of the GC and MS parameters is shown in the Supplementary Data.

**Figure 2:**
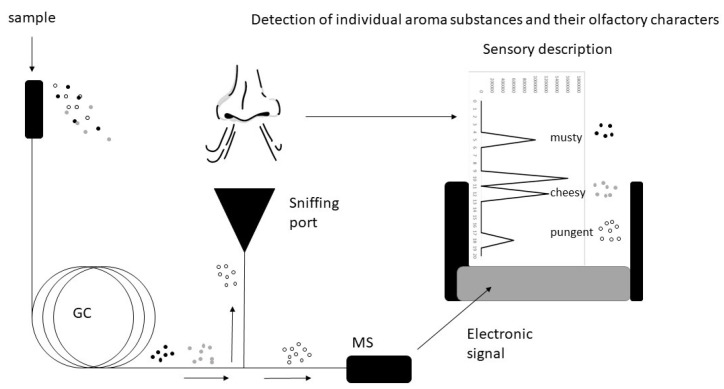
Overview of the gas chromatography–olfactometry system (modelled after [32]).

The GC method used in our experiments is a gas chromatography–olfactometry (GC/O) method. This combines information from the chemical characterization and the obtained odour data. The GC/O approach uses a GC-MS system combined with an olfactory detector port. At the end of the GC is a sniffer mask where trained panelists (employees of Symrise AG) can smell the gas and score information about the intensity. At the end of the GC column, after separation of the chemical compounds in the gas mixture, the sample is split and enters the MS detector and the panelist’s nose in equal parts (Figure 2).

The panelists sense the incoming fractions, whereby each time an odour is detected, a sensory response is given as to the presence and type of odour. Once an odour is detected by the panelist, a button is pressed and the odour is described. This provides an olfactogram that can be used to correlate the chemical information in the chromatogram with the sensory perceptions of the panelist. All tests were performed in triplicate at the end of the incubation time. 

### 2.3. Sensory Analysis

The fourteen panelists used for the free choice profiling were employees of Rhine-Waal University of Applied Sciences and were chosen at random in order to obtain consumer-related panel. At the beginning, a questionnaire was used to determine possible health conditions that could influence or exclude odour sensory detection. 

Each panelist received four selected samples, which were prepared according to the method described above (Section 2.1) and incubated for at least seven days. The tested combinations are listed in Table 3.

The samples were selected according to the preliminary tests in such a way that one sample had a particularly intense musty odour (MlShCj), two samples had a slightly musty odour (SeMoCj and MlSeSh) and one sample showed no musty odour (SeMoPa). In order to sensitise the panelists to the odour, regular training sessions were carried out. A panelist was considered trained after 5 training sessions. On the first day of testing, an individual attribute list was prepared by each tester using the simple descriptive test [33]. Here, the descriptions could be chosen freely from a given list or based on that list. Each panelist received individual test score sheets on the following test days, which were created based on the respective attribute list. Each attribute was provided with a scale on which panelists could mark intensities, with each panelist rating each sample three times. The individual test sheets created 14 individual datasets that resulted in a consensus configuration using translation, rotation, centering, and reflection using generalized Procrustes analysis. As individual test subject configurations were matched as closely as possible, variations between test subjects were eliminated and the datasets were made to be comparable [34,35,36,37,38]. The analysis was performed using the XLSTAT statistical tool for Microsoft Excel and included the Procrustes analysis of variance and the consensus configuration for principal component analysis [39].

### 2.4. Statistical Analysis

Statistics were performed using GraphPad Prism (GraphPad Software Inc., San Diego, CA, USA). Data were expressed as means (±standard deviation). Thus, statistically significant differences were assessed using multiple *t*-tests or two-way analysis of variance (ANOVA).

The colony-forming units (cfu/cm^2^) were investigated in surface culture on TSA (incubation at 37 °C for 24 h). Before and after incubation, the microbial count on the test swatches was determined in a similar manner. The number of colony-forming units (cfu/cm^2^) on plates was used to calculate the microbial load in the extraction liquid (c_wei_) (Equation (1)):

Equation (1): Weighted arithmetic average

(1) $$C_{wei}=\frac{\sum^{\;}C}{\left(n_{1}*1\right)+\left(n_{2}*0.1\right)}*d$$

where *C_wei_* = the weighted arithmetic average, ∑*C* = the sum of viable cell count of all agar plates used for calculation, *n*_1_ = the count of agar plates with the lowest evaluable dilution, *n*_2_ = the count of agar plates of the next higher dilution stage, and *d* = the dilution factor of the lowest evaluable dilution stage

To calculate the logarithmic reduction factor, the logarithmic cfu value of the biomonitors was subtracted from the logarithmic mean of the initial microbial counts of the respective biomonitors (Equation (2)).

Equation (2): Logarithmic reduction factor

(2) $$LR=K_{0}-K_{S}$$

where *LR* = the logarithmic reduction factor, *K*_0_ = the common logarithmic of the microbial count per mL of the initial load on the swatches before laundering, and *K_S_* = the common logarithmic of the microbial count per mL of the initial load on the swatches after laundering.

### 2.5. Evaluation of the Reduction of Microbial Counts after a Simulated Wash Cycle (Rotawash)

Studies [40,41] have shown that the Rotawash device is capable of simulating the main wash and rinse processes of a domestic washing machine. To analyze the effects of antibacterial substances that may reduce the bacterial numbers during and after laundering, samples were subjected to a laundering process according to the procedure described by Schages et al. [41]. The Rotawash device has 12 vessels, which allow the simultaneous measurement of different parameters. Each vessel was loaded with 8 steel beads. The time tested was 60 min, the temperature studied was 30 °C for the main wash with a water inlet temperature of approximately 15–20 °C, and the detergent used was benzalkonium chloride (0.4% and 0.8%).

The textiles were prepared as described in Section 2.1. After the Rotawash completed a run, the textiles were quantified by extraction with 1 mL TSB-TLH-thio (TSB with 30 g L^−1^ Tween 80, 0.3 g L^−1^ lecithin, 1 g L^−1^ histidine, 5 g L^−1^ sodium-thiosulfate). Tests were carried out in a 1.5 mL reaction tube (Sarstedt, Nümbrecht, Germany) for 10 min at 15 °C and 1000 rpm in an orbital incubating shaker (Thermomix comfort, Eppendorf, Hamburg, Germany). The colony-forming units (cfu/cm^2^) were investigated in surface culture on TSA for bacteria (incubation for 24 h at 37 °C). After laundering, the microbial counts on the test swatches were determined similarly.

An overview of the tested microorganism combinations is shown in Table 3. The combinations were selected on the basis of the preliminary tests in such a way that the samples with a particularly musty odour were tested in this study.

## 3. Results

The model developed for the in vitro formation of malodour was based on three influencing factors—the mixture of malodourous bacteria, the textile matrix, and a growth medium, which provided nutrients and humidity. Since it can be assumed that remaining body fats may serve as a major nutrient source, especially on textiles washed at low temperature, beef tallow was chosen as an ingredient that might simulate this phenomenon. 

For the selection of the test organisms, various preliminary tests were carried out. 

As shown in Table 3**,** different combinations of bacterial strains were cultivated together on a textile matrix with beef tallow and the formation of malodour was evaluated using a trained sniffer panel after three and seven days. While some combinations produced a strong malodour, others did not smell at all or just exhibited a weak or temporary malodour.

Based on these results, the combination of *M. luteus* + *S. hominis* and *C. jeikeium* (MlShCj) was selected, which was the only setup that showed strong and continuous malodour formation, even after three days. Moreover, this combination comprised a mixture of bacteria from different sources related to laundering. While *Micrococci* can routinely be found in washing machines and on worn textiles [4,42], *P. aeruginosa* has been isolated from washing machine biofilms [28,42] and *S. hominis* has been associated with the formation of body odour due to the formation of thioalcohols [43].

At the beginning, the microbial counts on the textile before and after the incubation period of 7 days were determined for MlShCj (see Table 4).

The results show that the bacterial count after incubation (t7) was about one-half logarithmic level higher than the bacterial count at the beginning of the incubation (t0). The microbial count was also determined only from the beef tallow in order to ensure that no bacteria were introduced into our experiment via these ingredients, however no microorganisms were found either before or after incubation (data not shown).

Moreover, the odour intensity was evaluated daily by the sniffer panel for a period of 14 days, showing that the highest intensity of malodour was reached after 7 days of incubation, while a longer incubation period did not lead to an increased intensity of malodour.

The volatile substances produced during growth of the MlChSj combination were also analysed by gas chromatography–olfactometry (GC/O). Subsequently, various bacterial combinations, including MlShCj, were given to a sniffer panel for qualitative evaluation (free choice profiling).

### 3.1. Gas Chromatograpy–Olfactometry (GC/O)

GC/O was used to identify the most perceived substances associated with wet fabric malodour as described below. In brief, the volatile compounds produced in the malodour model were separated by GC and sent to a sniffing port, where a trained sniffer evaluated the odours associated with each GC peak. Whenever a substance was sensed that resembled a wet-fabric-like malodour, this compound was identified via MS. A total overview of the identified substances per sample can be found in the Supplementary Data. The outcome of the GC/O analysis is shown in Table 5. The MlShCj sample was measured at elevated concentrations (five textile pieces were incubated together). 

The results show that DMDS, DMTS, and indole were associated strongly with a wet-fabric-like malodour. These substances have been described as cabbage-like (DMDS), moldy (DMTS), and technical (indole) [44]. Interestingly, the odour intensity did not always correlate with their qualitative contribution to the perceived malodour. DMDS and DMTS could only be slightly perceived olfactorily (intensity 1), whereas indole was rated as very intense (intensity 4). In addition, para-cresol, which was described as animal-like and urine-like, could be identified as a substance contributing to malodour and was detected with an intensity of 2. Finally, an unknown substance with the characteristic “wet fabric malodour” could be detected at a retention time of 15.12 and was rated as relatively dominant (intensity 4).

The respective concentrations of the peaks were calculated. Using the following equation, in which the peak area *C_A_* is the concentration as a percentage, a is the analyte and *i* is the number of other components:

$$C_{A},\%=\frac{A_{a}}{\sum_{i=1}^{n}A_{i}}\;\times \;100$$

Consequently, Table 6 shows the calculated concentrations of DMDS, DMTS, and indole for the ten combination measured. It can be seen that MlShCj shows the highest amounts of DMDS (0.36%) and DMTS (0.10%) compared to the other samples. Likewise, indole is present in this sample in a high amount, although the highest values can be seen for the SeMoPa sample.

### 3.2. Sensory Analysis—Free Choice Profiling

To further characterise the type and equality of the malodour produced by the model, the odour produced by MlShCj was described by a sniffer panel using free choice profiling.

**Figure 3:**
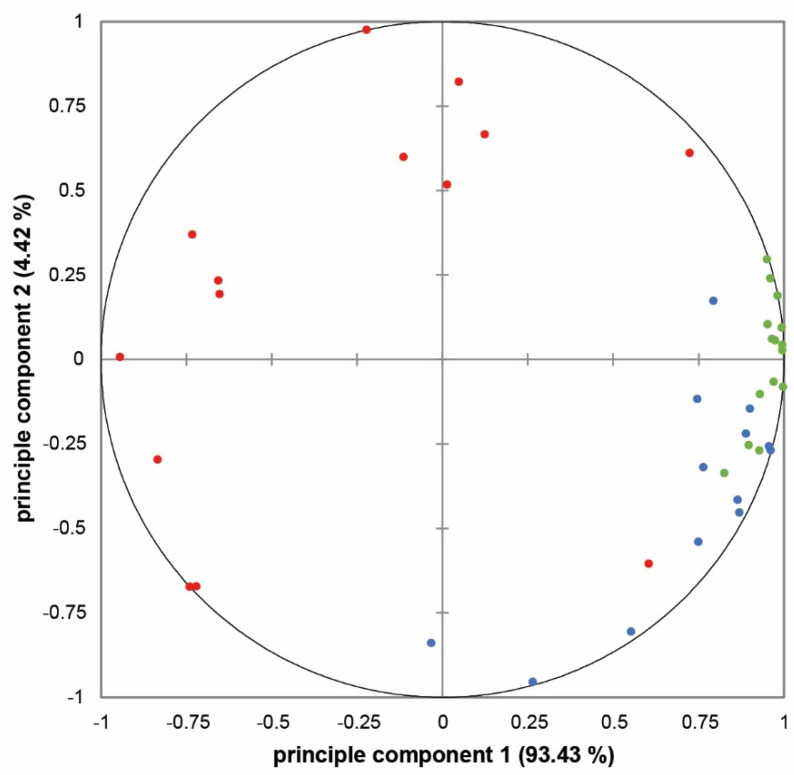
Principle component analysis (PCA) correlation circle: first and second principal components of odour samples (*n* = 14). Green = musty, blue = pungent, red = cheesy.

Figure 3 shows the principle component analysis correlation circle for the attributes’ ratings for the malodour samples produced by different bacterial combinations. The correlation between a variable and a principal component (PC) is used as the coordinates of the variables on the PC. The representation of the variables differs from the representation of the observations; observations are represented by their projections, however variables are represented by their correlations [45]. The correlation circle includes the first two principal components, which cover 93.43% and 4.42% of the data variance, respectively. It can be seen that the attributes musty (1) and pungent (0.886) determined the positive area of the first PC (93.43%, *x*-axis), while the negative area of the first PC was dominated by the attribute cheesy (−0.776). The positive and negative parts of the correlation circle show only whether there is a strong positive or negative correlation between the attributes. The second PC (4.42%, *y*-axis) was dominated by the attribute cheesy (0.577) in the positive range and by the attribute pungent (−0.400) in the negative range. Here, the location of the attribute musty was approximately 0. Furthermore, the distances of the attribute musty between the panelists were very small, indicating a distinct attribute. The distances of the attribute pungent between the panelists were slightly larger (three squares) and thus slightly less pronounced than the attribute musty. The attribute cheesy had very large distances between the panelists and was thus only slightly pronounced. Overall, the results show that there was a strong correlation between the attributes malodour and pungent, otherwise the attribute cheesy did not show direct correlations with the other attributes.

**Figure 4:**
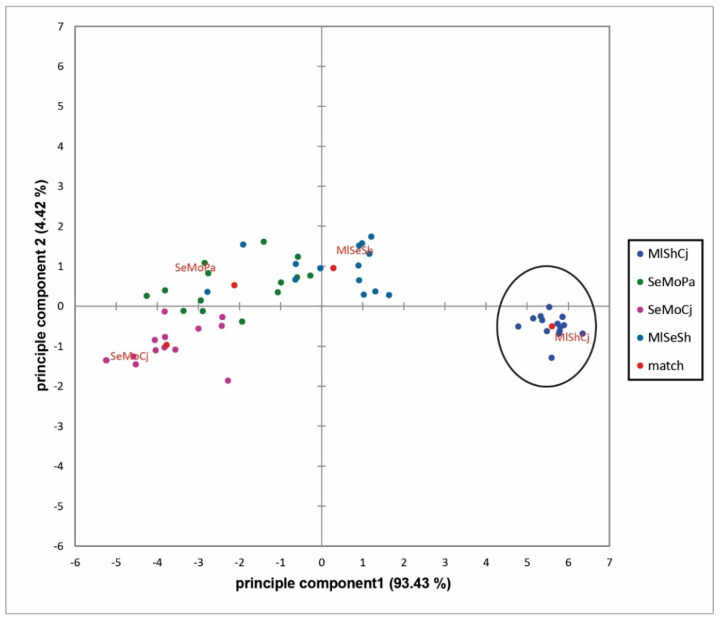
Object map of the samples (MlShCj = *M. luteus* + *S. hominis* + *C. jeikeium*; SeMoPa = *S. epidermidis* + *M. osloensis* + *P. aeruginosa*; SeMoCj = *S. epidermidis* + *M. osloensis* + *C. jeikeium*; MlSeSh = *M. luteus* + *S. epidermidis* + *S. hominis*).

Figure 4 shows the object map of the PCA for the different bacterial combinations. For the first principal component (*x*-axis), a cluster of the MlShCj sample can be seen in the far positive range with a match value of 5.6 (black border). In the neutral region, the MlSeSh sample is shown with a match value of 0.3. The negative region of the first principal component was determined by the samples SeMoPa (−2.1) and SeMoCj (−3.8). For the second principal component (*y*-axis), the agreement values were between −1 and 1. In the negative area the samples SeMoCj (−1.0) and MlShCj (−0.5) are shown, while in the positive area the samples SeMoPa (0.5) and MlSeSh (1.0) are shown. Overall, the results show that the MlShCj sample clearly clusters, whereas the other samples are relatively divergent from each other. Thus, the MlShCj sample was rated the same by the panelists, while the other samples were rated differently.

### 3.3. Bacterial Growth and Effects of Biocides

**Figure 5:**
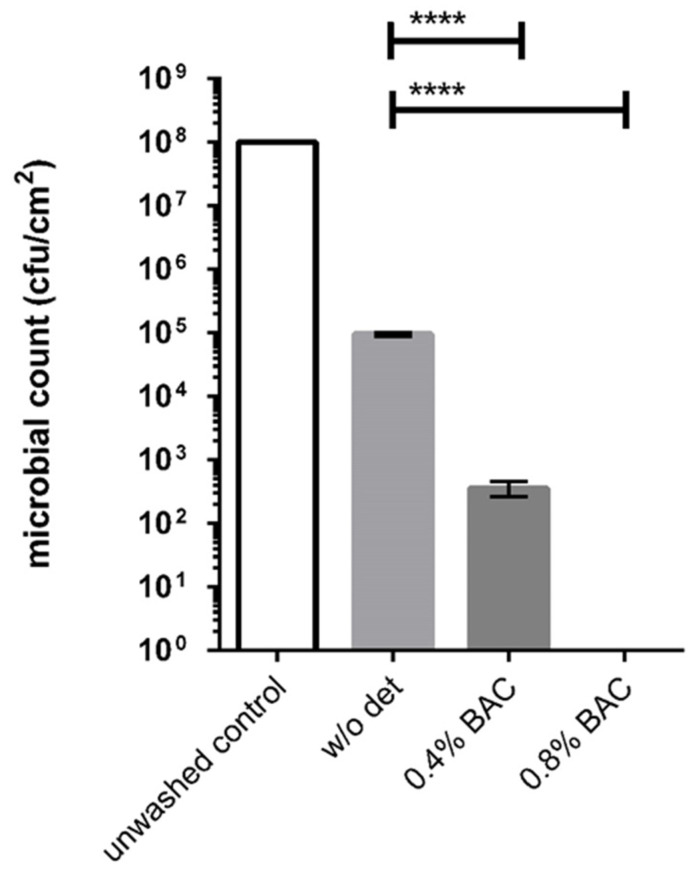
Microbial counts of MlShCj models after a simulated wash cycle (Rotawash) without detergent (w/o det), with 0.4% benzalkonium chloride (BAC) (0.4% BAC), and with 0.8% BAC (0.8% BAC), followed by a 7 day incubation period as described for the malodour model in general. An unwashed sample served as the control, ****: *p*< 0.0001.

Although the development of the described method focused on the formation of malodour, differences in the bacterial growth are likely to influence the quality and quantity of volatile substances. Since the method aimed to investigate the formation of textile malodour associated with slow or insufficiently dried laundry in order to analyse the effects of antibacterial substances, which may reduce the bacterial numbers during and after laundering, MlShCj samples were subjected to a laundering process according to the procedure described by Schages et al. (2020), followed by a 7 day incubation period as described above. At the end of the incubation time, logarithmic reduction factors were determined after a simulated wash cycle without detergent or with 0.4% or 0.8% benzalkonium chloride, as used in common hygienic rinse aids (Figure 5).

The results in Figure 5 show that there are significant differences in the microbial counts depending on whether the MlShCj model has undergone a wash process before incubation. For the unwashed control, a bacterial count of 1 × 10^8^ cfu/cm^2^ could be observed after 7 d incubation. In contrast, after a simulated wash cycle without detergent, a bacterial count 1 × 10^5^ cfu/cm^2^ was found, while after the addition of 0.4% BAC, only 4 × 10^2^ cfu/cm^2^ were present on the malodour model. When adding 0.8% BAC to the washing process, no bacteria were found afterwards.

The same trend was observed for the remaining odour; after the simulated wash cycle without detergent, a clear reduction of malodour could be detected, while with the addition of BAC (both 0.4% and 0.8%), no malodour could be detected by the panelists.

## 4. Discussion

### 4.1. Gas Chromatograpy–Olfactometry (GC/O)

The substances DMDS, DMTS, and indole have already been known to be responsible for wet-laundry-associated malodour [44]. However, the presence of 4-methyl-3-hexenoic acid, which has been attributed to this type of malodour before, could not be detected in this study. It cannot be excluded, however, that this substance was produced as well, since the database we used did not contain this compound.

The two sulfur-containing compounds DMDS and DMTS may possibly have formed via bacterial catabolism of proteins [46], which are present at approximately 0.8 g per 100 g in beef tallow [47].

Denawaka et al. identified six volatile compounds, including DMDS and DMTS, from dirty clothes before and after washing at low temperatures with a perfume-free powder detergent [19]. They examined worn socks and worn T-shirts using a static headspace–multicapillary column–gas chromatography–ion mobility spectrometry method. Here, DMDS and DMTS, among others, were detected in five out of eight sock samples. Another important finding of the study was that the volatile compounds were completely removed from the socks after prewashing (20 °C) with added detergent. In contrast, in the T-shirt samples, of the compounds identified in this study, only DMTS was found in four of nine samples. In addition, butyric acid, 2-heptanone, 2-octanone, and 2-nonanone were identified as other volatile compounds that are considered to have the potential to cause odour, which have already been described as human body odourants [48,49].

DMDS and DMTS were also identified as potential markers for textile-associated malodour in a study by Stapleton et al. in 2013 [50]. These results confirm the data generated in our study. In addition to the mentioned compounds, para-cresol was identified at a concentration of 0.10%. This substance is known to be a component of human sweat, which is particularly attractive to female mosquitoes [30,31]. Furthermore, para-cresol is considered to be a major component of pig odour [51]. It has already been demonstrated that para-cresol can be formed from the bacterial degradation of tyrosine [52,53]. Since small amounts of tyrosine are present in the beef tallow (27 mg per 100 g) [47], the MlShCj sample may form small amounts of para-cresol.

At a retention time of 15.12 min, the GC/O showed an unknown compound, which was rated as intense (intensity 3) by the sensory analysts and described as “wet fabric malodour” (cf. Table 5). Since this compound could not be identified due to a lack of database entries, further analysis of this compound might reveal its role in the composition of the malodour.

Although remaining partly artificial, the data derived from this model suggest that DMDS, DMTS, para-cresol, and indole might be important parts of the malodourous cocktail, which resembles a wet-dustcloth-like odour. In particular, the combination of *Micrococcus luteus*, *Staphylococcus hominis,* and *Corynebacterium jeikeium* was able to produce increased concentrations of DMDS and DMTS, which have already been identified as components in laundry malodour in previous studies [50].

### 4.2. Laundry Malodour Bacteria

Nix et al., who analysed the microbial communities in a domestic washing machine, were able to identify *Micrococcus luteus* as a frequent coloniser of the rubber seal and the detergent chamber [28]. Likewise, Callewaert et al. (2015) found *Micrococcus sp.* on worn cotton clothes [4], whereas Gattlen et al. in a culture-dependent approach could not isolate *Micrococci* from a washing machine but could isolate *Staphylococci* [42]. Moreover, Callewaert et al. (2015) suggested that skin-derived *Corynebacteria* and *Staphylococci* might be enriched on the textiles during laundering, while *Micrococci* will remain quite abundant [4]. In preliminary tests, we were able to show that the genera *Micrococcus* and *Staphylococcus* can frequently be found on textiles exhibiting wet-fabric-like malodour (unpublished results). Finally, *Corynebacteria* have been shown to be present on odourous textiles as well, although are mainly associated with sweaty odours [21,54,55,56]. Thus, the strains used for the malodour model in this study resemble entities that can frequently be found on textiles or in washing machines.

Unlike the suggestion made in some publications, it should be considered that laundry-associated malodour may not be produced by a single microbial strain. In 2014, Marmann et al. investigated the chemical diversity of terrestrial microorganisms using co-cultivation [57]. Based on various studies [58,59,60], it seems likely that the number of chemicals produced by microorganisms is many times higher than previously known. Many biosynthetic genes are obviously not active under normal laboratory conditions, with the study by Marmann et al. clearly showing that co-cultivation considerably increases the chemical diversity of secondary metabolites produced by bacteria. Likewise, it was shown that increased concentrations of indole and diketopiperazines were measured during co-cultivation of *B. thuringiensis* and *B. megaterium*, while co-cultivation of *B. megaterium* with another bacterial strain did not result in higher synthesis of the mentioned substances. Similar results were also found in other studies related to the co-cultivation of fungi and bacteria [61,62].

As mentioned before, it should, thus, be questioned if only one species can be responsible for the formation of wet-fabric-like malodour. For example, Kubota et al. (2012) identified *Moraxella osloensis* as the main species responsible for the formation of this type of malodour in Japan [8]. In contrast, our results suggest that *Moraxella osloensis* might not be the only strain that is related to the formation of wet fabric malodour.

It should be mentioned that the method described here uses cotton as the basis for the experiments. Previous studies have already shown that the fibre content is a major factor in residual body odour [63,64] and it has been demonstrated that there is a difference between cotton and polyester in terms of malodour [64,65]. A future consideration of the developed model with different fibre contents would, therefore, be desirable and appropriate. Besides the influence of different textiles, the type of drying could also have an influence on the formation of malodour. A recent study by Pugliese et al. showed that the odour of fresh laundry is formed during linen drying and under the influence of UV [66].

### 4.3. Sensory Analysis—Free Choice Profiling

Although the use of GC is an important way to identify substances that are part of the malodourous cocktail, the model aimed to investigate the formation of laundry-associated malodour from a consumer perspective. Since it must be assumed that a single substance is not responsible if textiles are considered malodourous, the model should predominately be able to simulate a “malodour” from a consumer’s perspective.

To follow this approach, the samples were analyzed using free choice profiling, which showed significant differences between the four samples tested. On the one hand, Figure 4 shows that the profile for MlShCj was clearly separated from the other samples, which means that it is different in its characteristics. On the other hand, the figure shows that the panelists rated this sample as best at simulating a musty and pungent textile malodour.

The fact that not all panelists may use the attributes in the same way makes it difficult to assess the correlation between the principal components and the original attributes, so the use of a correlation circle is necessary [67]. Since the information for a variable in the correlation circle grows as the correlation coefficient increases [68], it is recommended that only correlation coefficients ≥0.5 and ≤−0.5 be considered. Except for individual panelists, this applies to all three of the attributes described in this study (see Figure 3). The correlation circle shows that almost all attributes are close to the edge and have very high correlation coefficients. Furthermore, it can be seen that all quotations of the term on the right-hand, positive side of the first principal component correlate strongly with each other. This suggests that all panelists understood the terms “musty” and “pungent” in the same way. In contrast, the ratings for the term “cheesy” are scattered over three squares of the correlation circle, which means that that this attribute was not understood in an equal manner.

By combining different bacterial strains, it could clearly be shown that a combination of different microorganisms might be necessary for the formation of strong malodour, although single microorganisms can also produce a slight malodour (see Table 3) The most intense odours would be identified in combinations. Indeed, a combination of *Micrococcus luteus*, *Staphylococcus hominis*, and *Corynebacterium jeikeium* embedded in beef tallow turned out to be especially suitable for producing the malodour, which was considered particularly musty and pungent and was shown to produce odourous substances typical of a wet-fabric-like malodour, as confirmed by GC/O. As shown in this study, the model is also suitable for investigating interventional means; for instance, malodour formation could be inhibited by a prior laundering process using antibacterial ingredients. Although bacterial growth was not inhibited completely under all tested conditions, it must be reasoned that a significant antibacterial effect during laundering can prevent malodour formation. Since wash temperatures are likely to further decrease in the future, malodour might be found more often, and the model developed here may help to develop suitable counteractions.

## Figures and Tables

**Table 1:** Bacterial test strains.

Strains	Code
*Corynebacterium jeikeium*	DSM 7171, ATCC 43734
*Micrococcus luteus*	DSM 1790, ATCC 10240
*Moraxella osloensis*	DSM 6998, ATCC 19976
*Pseudomonas aeruginosa*	DSM 939, ATCC 15442
*Staphylococcus epidermidis*	DSM 1798, ATCC 12228
*Staphylococcus hominis*	DSM 20329, ATCC 27845

**Table 2:** Important gas chromatographic parameters.

Program Step/Device Setting	Parameter
Oven program	- 50 °C for 0 min, then 8 °C/min to 230 °C for 22.5 min - Cycle time: 45 min
TDU tube	- 1.0726 bar
Universal injector	- Cryo cooling: −30 °C (using liquid nitrogen)
Desorption of the TDU tube	- Heat up to 280 °C - Holding time: 6 min
Mass spectrometer	- Low mass: 33.0 - High mass: 300.0 - Threshold: 150

**Table 3:** Overview of the bacterial combinations used for the tests (*n* = 5). Intensities ranged from 0 (no odour) to 3 (intensive malodour).

Combination of Microorganisms	Abbreviation	Evaluation after Three Days	Evaluation after Seven Days
*M. luteus + S. epidermidis + M. osloensis*	MlSeMo	0	1
*M. luteus + S. epidermidis + S. hominis*	MlSeSh	0	3
*M. luteus + S. epidermidis + P. aeruginosa*	MlSePa	0	2
*M. luteus + S. epidermidis + C. jeikeium*	MlSeCj	0	2
*M. luteus + M. osloensis + S. hominis*	MlMoSh	2	0
*M. luteus + M. osloensis + P. aeruginosa*	MlMoPa	0	0
*M. luteus + S. hominis + C. jeikeium*	MlShCj	3	3
*M. luteus + P. aeruginosa + C. jeikeium*	MlPaCj	1	1
*S. epidermidis + M. osloensis + S. hominis*	SeMoSh	2	1
*S. epidermidis + M. osloensis + P. aeruginosa*	SeMoPa	2	2
*S. epidermidis + M. osloensis + C. jeikeium*	SeMoCj	2	2
*S. epidermidis + S. hominis + C. jeikeium*	SeShCj	1	2
*S. epidermidis + P. aeruginosa + C. jeikeium*	SePaCj	1	1
*M. osloensis + S. hominis + P. aeruginosa*	MoShPa	0	1
*M. osloensis + P. aeruginosa + C. jeikeium*	MoPaCj	0	0
*S. hominis + P. aeruginosa + C. jeikeium*	ShPaCj	0	0
*M. luteus*	ML	1	2
*M. osloensis*	Mo	1	2
*P. aeruginosa*	Pa	0	0
*C. jeikeium*	Cj	0	0
*S. hominis*	Sh	0	0
*S. epidermidis*	Se	0	0
Control		0	1

**Table 4:** Microbial counts on the sample (textile) before and after incubation.

Sample	Microbial Count (cfu/cm^2^)
MlShCj before incubation	4.15 × 10^7^
MlShCj after incubation	1.04 × 10^8^
Control (beef tallow only)	0

**Table 5:** GC/O results for the MlShCj sample. Intensity range: 1 = weak to 4 = very strong; n.A. = not analysed.

Retention Time (min)	Identified Substance	Intensity	Odour Description
2.62	n.A.	1	fatty, waxy
3.67	n.A.	1	mouldy
4.07	Dimethyl Disulphide	1	cabbage
5.88	n.A.	1	acidic, penetrative
6.61	n.A.	1	acidic, mouldy
7.65	n.A.	1	acidic, waxy
8.19	Dimethyl Trisulphide	1	mouldy, cabbage
8.86	n.A.	3	mouldy, fatty, rotten
10.02	n.A.	2	fatty, meaty, metalic
10.48	n.A.	2	fatty, green, cucumber, aldehyde
12.89	n.A.	1	fatty, meaty
14.72	n.A.	1	animalic, scratchy, urine
15.12	n.A.	3	acidic, fatty, wet fabric malodour
17.07	n.A.	1	roasted, caramel
17.72	p-Cresol	2	animalic, urine
17.98	n.A.	1	powdery, scratchy
18.49	n.A.	1	acidic, fatty
18.90	n.A.	2	technical, phenolic
21.85	Indole	4	technical Indole
22.31	n.A.	3	phenolic, technical, like Indole
23.23	n.A.	2	technical, acidic
23.62	n.A.	1	sweet, phenylic, honey, fruity
26.78	n.A.	2	phenolic, smokey, burnt
26.96	n.A.	2	phenolic, smokey, burnt
28.60	n.A.	2	mouldy, acidic

**Table 6:** Concentrations of dimethyl disulfide, dimethyl trisulfide, and indole in the measured samples.

Sample	Dimethyl Disulphide (%)	Dimethyl Trisulfide (%)	Indole (%)
MlShCj	0.36	0.10	0.24
MlSeCj	0.08	0.00	0.17
SeMoPa	0.07	0.00	0.56
MlSeSh	0.04	0.01	0.12
SeShCj	0.06	0.00	0.07
MoShPa	0.03	0.00	0.23
Ml	0.05	0.01	0.13
Mo	0.02	0.00	0.03
SeMoCj	0.04	0.02	0.15
ShPaCj	0.02	0.00	0.13

## Supplementary Materials

The following are available online at https://www.mdpi.com/article/10.3390/microorganisms9050974/s1: Table S1: Detailed summary of substances found on the samples. Table S2: Detailed summary of GC and MS parameters.

## References

[1] Bockmühl D.P., Schages J., Rehberg L. (2019). Laundry and textile hygiene in healthcare and beyond. Microb. Cell.

[2] Bockmühl D. (2017). Laundry hygiene-how to get more than clean. J. Appl. Microbiol..

[3] Munk S., Johansen C., Stahnke L.H., Adler-Nissen J. (2001). Microbial survival and odor in laundry. J. Surfactants Deterg..

[4] Callewaert C., Van Nevel S., Kerckhof F.-M., Granitsiotis M.S., Boon N. (2015). Bacterial Exchange in Household Washing Machines. Front. Microbiol..

[5] Van Herreweghen F., Amberg C., Marques R., Callewaert C. (2020). Biological and Chemical Processes that Lead to Textile Malodour Development. Microorganism.

[6] Nagoh Y., Tobe S., Watanabe T., Mukaiyama T. (2005). Analysis of Odorants Produced from Indoor Drying Laundries and Effects of Enzyme for Preventing Malodor Generation. Tenside Surfactants Deterg..

[7] Takeuchi K., Hasegawa Y., Ishida H., Kashiwagi M. (2011). Identification of novel malodour compounds in laundry. Flavour Fragr. J..

[8] Kubota H., Mitani A., Niwano Y., Takeuchi K., Tanaka A., Yamaguchi N., Kawamura Y., Hitomi J. (2012). Moraxella Species Are Primarily Responsible for Generating Malodor in Laundry. Appl. Environ. Microbiol..

[9] Grice E.A., Segre J.A. (2011). The skin microbiome. Nat. Rev. Microbiol..

[10] Costello E.K., Lauber C.L., Hamady M., Fierer N., Gordon J.I., Knight R. (2009). Bacterial Community Variation in Human Body Habitats Across Space and Time. Science.

[11] Cundell A.M. (2018). Microbial Ecology of the Human Skin. Microb. Ecol..

[12] Troccaz M.C., Gaïa N., Beccucci S., Schrenzel J., Cayeux I., Starkenmann C., Lazarevic V. (2015). Mapping axillary microbiota responsible for body odours using a culture-independent approach. Microbiome.

[13] James A.G., Austin C.J., Cox D.S., Taylor D., Calvert R. (2012). Microbiological and biochemical origins of human axillary odour. FEMS Microbiol. Ecol..

[14] Fredrich E., Barzantny H., Brune I., Tauch A. (2013). Daily battle against body odor: Towards the activity of the axillary microbiota. Trends Microbiol..

[15] Natsch A., Gfeller H., Gygax P., Schmid J., Acuna G. (2003). A Specific Bacterial Aminoacylase Cleaves Odorant Precursors Secreted in the Human Axilla. J. Biol. Chem..

[16] Chung H., Seok H.J. (2012). Populations of malodor-forming bacteria and identification of volatile components in triolein-soiled cotton fabric. Fibers Polym..

[17] Teufel L., Pipal A., Schuster K., Staudinger T., Redl B. (2010). Material-dependent growth of human skin bacteria on textiles investigated using challenge tests and DNA genotyping. J. Appl. Microbiol..

[18] Hammond C.J. (2013). Chemical composition of household malodours*—*An overview. Flavour Fragr. J..

[19] Denawaka C.J., Fowlis I.A., Dean J.R. (2016). Source, impact and removal of malodour from soiled clothing. J. Chromatogr. A.

[20] Stapleton K., Dean J.R. (2013). A preliminary identification and determination of characteristic volatile organic compounds from cotton, polyester and terry-towel by headspace solid phase microextraction gas chromatography–mass spectrometry. J. Chromatogr. A.

[21] Callewaert C., De Maeseneire E., Kerckhof F.-M., Verliefde A., Van De Wiele T., Boon N. (2014). Microbial Odor Profile of Polyester and Cotton Clothes after a Fitness Session. Appl. Environ. Microbiol..

[22] McQueen R.H., Laing R.M., Brooks H.J.L., Niven B.E. (2007). Odor Intensity in Apparel Fabrics and the Link with Bacterial Populations. Text. Res. J..

[23] Honisch M., Stamminger R., Bockmühl D. (2014). Impact of wash cycle time, temperature and detergent formulation on the hygiene effectiveness of domestic laundering. J. Appl. Microbiol..

[24] Honisch M., Brands B., Weide M., Speckmann H.-D., Stamminger R., Bockmühl D.P. (2016). Antimicrobial Efficacy of Laundry Detergents with Regard to Time and Temperature in Domestic Washing Machines. Tenside Surfactants Deterg..

[25] Honisch M., Stamminger R., Bockmühl D. (2016). Impact of Time and Temperature on the Inactivation of Microorganisms in Domestic Washing Machines. J. Appl. Microbiol..

[26] Lucassen R., Merettig N., Bockmühl D.P. (2013). Antimicrobial Efficacy of Hygiene Rinsers under Consumer-Related Conditions. Tenside Surfactants Deterg..

[27] Bloomfield S.F., Exner M., Signorelli C., Scott E.A. (2013). Effectiveness of Laundering Processes Used in Domestic (Home) Settings.

[28] Nix I.D., Frontzek A., Bockmühl D.P. (2015). Characterization of Microbial Communities in Household Washing Machines. Tenside Surfactants Deterg..

[29] Payne J., Kudner D. (1996). A Durable Antoodor Finish for Cotton Textiles. Text. Chem. Color..

[30] Linley J.R. (1989). Laboratory tests of the effects of p-cresol and 4-methylcyclohexanol on oviposition by three species of Toxorhynchites mosquitoes. Med. Veter. Èntomol..

[31] Hallem E.A., Fox A.N., Zwiebel L.J., Carlson J.R. (2004). Mosquito receptor for human-sweat odorant. Nature.

[32] D-NOSES_EU (2021). Gas Chromatography Olfactometry. https://odourobservatory.org/measuring-odour/gas-chromatography-olfactometry/.

[33] Deutsches Institut für Normung (2014). Sensorische Prüfverfahren—Einfach Beschreibende Prüfung.

[34] Lawless H., Klein B., Dekker M. (1991). Sensory Science Theory and Applications in Foods.

[35] Oreskovich D., Klein B., Sutherland J., Dekker M. (1991). Procrustes Analysis and it‘s Application to Free-Choice and other sensory profiling. Sensory Science Theory and Applications in Foods.

[36] Dijksterhuis G. (1995). Multivariate data analysis in sensory and consumer science: An overview of developments. Trends Food Sci. Technol..

[37] Lawless H., Heymann H. (2010). Sensory Evaluation of Food. Principles and Practices.

[38] Narain C., Paterson A., Reid E. (2004). Free choice and conventional profiling of commercial black filter coffees to explore consumer perceptions of character. Food Qual. Prefer..

[39] Addinsoft (2019). XLSTAT Statistical and Data Analysis Solution.

[40] Clarke J., Oakes L., Miller L., Hindley P., McGeechan P., Petkov J., Bockmühl D. (2018). Towards a Lab-Scale Efficacy Test Method for the Evaluation of Hygienic Laundry Rinse-Stage Disinfectants. Tenside Surfactants Deterg..

[41] Schages J., Stamminger R., Bockmühl D.P. (2020). A New Method to Evaluate the Antimicrobial Efficacy of Domestic Laundry Detergents. J. Surfactants Deterg..

[42] Gattlen J., Amberg C., Zinn M., Mauclaire L. (2010). Biofilms isolated from washing machines from three continents and their tolerance to a standard detergent. Biofouling.

[43] (2015). Microbiology S for G. Bacterial Genetic Pathway Involved in Body Odor Production Discovered. ScienceDaily. https://www.sciencedaily.com/releases/2015/03/150330213947.htm.

[44] Symrise A.G. (2019). Persönliche Kommunikation.

[45] Abdi H., Williams L.J. (2010). Principal component analysis. Wiley Interdiscip. Rev. Comput. Stat..

[46] Legrum W. (2015). Riechstoffe, Zwischen Gestank und Duft: Vorkommen, Eigenschaften und Anwendung von Riechstoffen und Deren Gemischen.

[47] Bundeszentrale für Gesundheit und Verbraucherschutz (2019). Bundeslebensmittelschlüssel.

[48] Garrido-Delgado R., Arce L., Guamán A., Pardo A., Marco S., Valcarcel M. (2011). Direct coupling of a gas-liquid separator to an ion mobility spectrometer for the classification of different white wines using chemometrics tools. Talanta.

[49] Zamora D., Alcalà M., Blanco M. (2011). Determination of trace impurities in cosmetic intermediates by ion mobility spectrometry. Anal. Chim. Acta.

[50] Stapleton K., Hill K., Day K., Perry J., Dean J. (2013). The potential impact of washing machines on laundry malodour generation. Lett. Appl. Microbiol..

[51] Borrel B. (2009). Why Study Pig Odor?. https://www.scientificamerican.com/article/why-study-pig-odor/.

[52] Mathus T. (1995). Anaerobic biogenesis of phenol and p-cresol from ρ-tyrosine. Fuel.

[53] Saito Y., Sato T., Nomoto K., Tsuji H. (2018). Identification of phenol- and p-cresol-producing intestinal bacteria by using media supplemented with tyrosine and its metabolites. FEMS Microbiol. Ecol..

[54] Natsch A., Gfeller H., Gygax P., Schmid J. (2005). Isolation of a bacterial enzyme releasing axillary malodor and its use as a screening target for novel deodorant formulations1. Int. J. Cosmet. Sci..

[55] McQueen R.H., Laing R.M., Wilson C.A., Niven B.E., Delahunty C.M. (2007). Odor Retention on Apparel Fabrics: Development of Test Methods for Sensory Detection. Text. Res. J..

[56] Li M., Budding A.E., Van Der Lugt-Degen M., Du-Thumm L., Vandeven M., Fan A. (2019). The influence of age, gender and race/ethnicity on the composition of the human axillary microbiome. Int. J. Cosmet. Sci..

[57] Marmann A., Aly A.H., Lin W., Wang B., Proksch P. (2014). Co-Cultivation—A Powerful Emerging Tool for Enhancing the Chemical Diversity of Microorganisms. Mar. Drugs.

[58] Knight V., Sanglier J.-J., DiTullio D., Braccili S., Bonner P., Waters J., Hughes D., Zhang L. (2003). Diversifying microbial natural products for drug discovery. Appl. Microbiol. Biotechnol..

[59] Brakhage A.A., Schuemann J., Bergmann S., Scherlach K., Schroeckh V., Hertweck C. (2008). Activation of fungal silent gene clusters: A new avenue to drug discovery. Nat. Compd. Drugs.

[60] Brakhage A.A., Schroeckh V. (2011). Fungal secondary metabolites*—*Strategies to activate silent gene clusters. Fungal Genet. Biol..

[61] Schroeckh V., Scherlach K., Nützmann H.-W., Shelest E., Schmidt-Heck W., Schuemann J., Martin K., Hertweck C., Brakhage A.A. (2009). Intimate bacterial-fungal interaction triggers biosynthesis of archetypal polyketides in Aspergillus nidulans. Proc. Natl. Acad. Sci. USA.

[62] Ola A.R.B., Thomy D., Lai D., Brötz-Oesterhelt H., Proksch P. (2013). Inducing Secondary Metabolite Production by the Endophytic Fungus Fusarium tricinctum through Coculture with Bacillus subtilis. J. Nat. Prod..

[63] McQueen R.H., Vaezafshar S. (2020). Odor in textiles: A review of evaluation methods, fabric characteristics, and odor control technologies. Text. Res. J..

[64] Abdul-Bari M.M., McQueen R.H., De La Mata A.P., Batcheller J.C., Harynuk J.J. (2020). Retention and release of odorants in cotton and polyester fabrics following multiple soil/wash procedures. Text. Res. J..

[65] Munk S., Münch P., Stahnke L., Adler-Nissen J., Schieberle P. (2000). Primary odorants of laundry soiled with sweat/sebum: Influence of lipase on the odor profile. J. Surfactants Deterg..

[66] Pugliese S., Jespersen M.F., Pernov J.B., Shenolikar J., Nygaard J., Nielsen O.J., Johnson M.S. (2020). Chemical analysis and origin of the smell of line-dried laundry. Environ. Chem..

[67] Dijksterhuis G. (1996). Procrustes Analysis in Sensory Research.

[68] Meilgaard M.C., Carr B.T. (2006). Sensory Evaluation Techniques.

